# Reduced Fas ligand-expressing splenic CD5^+ ^B lymphocytes in severe collagen-induced arthritis

**DOI:** 10.1186/ar2795

**Published:** 2009-08-25

**Authors:** Steven K Lundy, David A Fox

**Affiliations:** 1 Department of Internal Medicine, Division of Rheumatology, University of Michigan Medical School, 4043 Biomedical Sciences Research Building, Ann Arbor, MI 48109-2200, USA; 2 Rheumatic Diseases Core Center, University of Michigan Medical School, 3918 Taubman Medical Center, Ann Arbor, MI 48109-5358, USA

## Abstract

**Introduction:**

The objective was to study immune regulation in a mouse model of rheumatoid arthritis that exhibits considerable heterogeneity of disease activity.

**Methods:**

T-cell receptor transgenic mice, in which nearly all CD4^+ ^T cells recognize a single peptide of type II collagen, were immunized with collagen and observed for development of arthritis for 4 weeks. At 28 days post-immunization, splenocytes were analyzed by flow cytometry and *in vitro *assays for markers of immune activation and regulation.

**Results:**

Disease severities ranging from 0 to 12 (on a 12-point scale) were observed. Among splenic lymphocyte populations, only the CD5^+ ^B-cell subset displayed a decrease in relative numbers as arthritis severity increased. Splenic CD5^+ ^B cells expressed higher levels of Fas ligand (FasL) than did CD4^+ ^T cells or CD5^- ^B cells in all mice, and antigen-dependent T-cell death correlated with higher levels of CD5^+ ^B cells in cocultures. Ratios of interleukin (IL)-17 to interferon-gamma production were higher in antigen-driven cultures of splenocytes from severely arthritic mice compared to mildly or nonarthritic mice. A correlation was established between the reduced production of IL-17 in antigen-driven T-cell/B-cell cocultures and FasL, but not IL-10. Confirmation of the direct killing effect of B cells on T cells was demonstrated using an antigen-specific T hybridoma cell line.

**Conclusions:**

Reduced numbers of splenic FasL^+ ^CD5^+ ^B cells correlated with increasing arthritis severity and decreased T-cell death in a T-cell receptor transgenic mouse model of collagen-induced arthritis. These 'killer' B cells may provide a novel mechanism for inducing T-cell death as a treatment for arthritis.

## Introduction

Rheumatoid arthritis (RA) is a destructive inflammatory disease of the joints, is associated with genetic and environmental risk factors, and shows strong evidence of being mediated by cells of the immune system [1,2]. The most important genetic risk factor for RA is the inheritance of polymorphic major histocompatibility complex (MHC) class II alleles containing the 'shared epitope', which occurs in approximately 40% of the healthy Caucasian population but in approximately 70% of RA patients within that group [1,3]. The critical role of MHC class II in the presentation of antigens to CD4^+ ^T helper (Th) lymphocytes has long implicated Th cells as effector cells in RA [4,5]. Evidence from animal models of arthritis has shown that Th cells, particularly Th17 cells, which produce the proinflammatory cytokine interleukin (IL)-17, play an important role in mediating arthritis [6-9]. The role of Th17 cells in human RA is less established, but it is known that IL-17 can act directly on fibroblast-like synoviocytes, macrophages, and osteoclasts to induce proinflammatory mediators implicated in RA synovial damage [10-12]. Thus, regulation of Th17 cells has become an attractive target for treatment of RA.

One level of control of Th cells is at the induction phase, which is regulated by direct interaction of naïve Th cells with professional antigen-presenting cells (APCs), including dendritic cells, activated macrophages, and activated B lymphocytes. The cytokine environment at the time of interaction between APCs and T cells drives Th cells to become Th1, Th2, regulatory T (Treg), or Th17 cells that produce the characteristic cytokines: interferon-gamma (IFNγ), IL-4, IL-10, or IL-17, respectively [13,14]. In mice, the presence of IL-6 and low levels of transforming growth factor-beta at the time of T-cell activation drives Th17 cell differentiation, whereas the presence of other Th cell cytokines such as IL-4 and IFNγ inhibits Th17 cell induction [15,16]. Interactions between co-stimulatory or regulatory cell surface molecules on APCs and their counter-receptors on T cells may also play an important role in Th cell cytokine production. Manipulating the cytokine microenvironement and/or interactions between APCs and Th cells may have profound effects on Th cell activation and disease outcome.

One level of control of Th cell activity involves the induction of Th cell death (apoptosis), which can be mediated through intrinsic or extrinsic pathways. The best studied extrinsic pathway of T-cell death involves ligation of the Fas death receptor (CD95) by Fas ligand (FasL, CD95L, or CD178) [17] and is dependent on activation of the target cell. Deficiencies in Fas or FasL lead to severe lymphoproliferative disease in mice, manifesting in a lupus-like condition [18]. Conversely, adoptive transfer of genetically engineered APCs with forced expression of FasL was shown to prevent arthritis in an animal model [19]. Previous studies have shown that FasL can be naturally expressed by mouse or human B cells and can lead to induction of Th cell apoptosis and control of inflammation [20-22]. In particular, the small population of CD5^+ ^B cells in the spleen has been shown to be constitutively positive for FasL and were potent mediators of T-cell apoptosis [23]. FasL expression on CD5^+ ^B cells was upregulated by IL-4 and IL-10, and previous studies have shown that IL-10 can be produced by CD5^+ ^B cells [23-25]. These results led us to hypothesize that CD5^+ ^B cells could play a role in regulating arthritis through interactions with arthritogenic T cells.

In the present study, we have used a T-cell receptor (TCR) transgenic (Tg) mouse model in which immunization with type II collagen (cII) can potentially stimulate all of the Th cells within the mouse. Initial results indicated that a group of these immunized cII TCR Tg mice developed severe arthritis with a rapid onset whereas other mice developed moderate, mild, or even no arthritis over a 28-day time period. Analysis of the splenic lymphocytes in these mice revealed that severely arthritic mice had relatively fewer CD5^+ ^B cells than mildly or moderately arthritic mice. FasL expression was highest on CD5^+ ^B cells compared with other lymphocyte subsets, but the level of expression on individual cells was not correlated to disease activity. Importantly, increased T-cell apoptosis correlated with the relative number of FasL^+ ^B cells in cocultures, and differences in IL-17 and IFNγ production patterns were evident after neutralizing FasL. A direct killing effect of B cells on a T-cell line specific for an arthritogenic autoantigen was also demonstrated. This is the first evidence that killer B cells may play a role in regulating arthritogenic T cells.

## Materials and methods

### Mice, immunizations, and arthritis measurement

Wild-type DBA/1LacJ mice and a breeding pair of TCR Tg (D1Lac.Cg-Tg(TCRa,TCRb)24Efro/J) mice were purchased from The Jackson Laboratory (Bar Harbor, ME, USA). Breeding was performed in the facilities of the Unit for Laboratory Animal Medicine of the University of Michigan Medical School, and all procedures were approved by the Institutional Animal Care and Use Committee. Transgene expression was confirmed in all offspring by polymerase chain reaction analysis of tail biopsies. The TCR transgene is reported to be specific for an immunodominant peptide of cII (cII_260-267_) when bound to MHC class II I-A^q ^molecules (DBA/1 background) [26]. C57BL/6- [Tg] DR-4- [KQ]ABB (human class II MHC DRB1*0401 [DR4] Tg) mice were purchased from Taconic (Hudson, NY, USA). Female mice were immunized at the base of the tail with 0.1 mg of freshly solubilized type II chicken collagen (Chondrex, Redmond, WA, USA) emulsified in complete Freund's adjuvant (CFA) (Chondrex). Paw swelling was assessed every 3 or 4 days following immunization and was scored on a 4-point scale for each paw: 0 = no arthritis, 1 = swelling and redness confined to digits, 2 = minor swelling and redness spreading from the digits to the distal paw, and 3 = major swelling and redness extending proximally from the paw. Mice were sacrificed on day 28 after immunization unless otherwise noted, and spleens were removed for processing of single-cell suspensions from individual mice. For some analyses, the splenocytes were pooled from mice with similar disease severity as follows: score of 0 to 2 = mild or no arthritis, score of 3 to 6 = moderate arthritis, and score of 7 to 12 = severe arthritis.

### Antibodies and flow cytometry

Anti-mouse CD19-allophycocyanin (clone 1D3), anti-mouse CD3-biotin (145-2C11), anti-mouse CD5-biotin (53-7.3), anti-mouse FasL-phycoerythrin (anti-mouse FasL-PE) (MFL3), anti-CD4-PE (H129.19), anti-CD8-fluorescein isothiocyanate (anti-CD8-FITC) (53-6.7), anti-CD38-FITC (clone 90), streptavidin-PE cychrome 7 conjugate (streptavidin-PECy7), and Annexin-V-FITC were purchased from BD Biosciences (San Jose, CA, USA). Propidium iodide (PI) was purchased from Sigma-Aldrich (St. Louis, MO, USA). Freshly isolated single-cell suspensions from mouse spleens were stained with purified FcBlock (2.4G2; BD Biosciences) before being labeled with anti-mouse CD19-APCs and either anti-CD5-biotin or anti-CD3-biotin for 30 minutes at 4°C in phosphate-buffered saline (PBS) containing 0.2% bovine serum albumin and 0.1% sodium azide. Cells were then washed in PBS and fixed in 1% paraformaldehyde overnight. Fixation solution was washed away thoroughly followed by labeling of the CD5-stained group with streptavidin-PECy7, anti-FasL-PE, and anti-CD38-FITC. Gating used for CD5^+ ^T and B cells was determined using magnetic bead-separated T and B cells and reflects the fact that CD5 expression on B cells is low in comparison with T cells. The few CD5^hi^CD19^+ ^cells on the dot plots that were excluded by gating are not present in the separated populations and most likely represent doublets of T and B cells as further indicated by higher forward scatter characteristics. The CD3-biotin-stained group was labeled with streptavidin-PECy7, anti-CD4-PE, and anti-CD8-FITC. Flow cytometry was performed on a Cytomics FC 500 flow cytometer (Beckman Coulter, Fullerton, CA, USA), and cell marker analysis was done using FlowJo software (TreeStar Inc., Ashland, OR, USA).

### Magnetic bead separation

Single-cell suspensions from pooled spleens of mice with severe, moderate, or mild arthritis were counted and washed in manufacturer-suggested buffer prior to labeling with anti-mouse CD19-coated magnetic microspheres (Miltenyi Biotec, Auburn, CA, USA). Cells were then passed over magnetized LS columns with the flow-through being collected as the B cell-depleted (T cell-enriched, approximately 80% T cells) fraction and the column retentate collected as the purified B-cell fraction (purity greater than 98% for all groups as determined by flow cytometry).

### Cell cultures for apoptosis measurements

For comparison of T-cell apoptosis in mice with severe, moderate, and mild arthritis, T cell-enriched fractions of pooled mouse splenocytes were cultured alone or at a 1:1 ratio with purified B cells in the absence or presence of 10 μg/mL of a synthetic peptide of cII, cII_259-273_, which encompasses the cII_260-267 _epitope recognized by the transgene (Peptide Synthesis Facility, University of Michigan). Cells were collected on day 7 of coculture and analyzed for apoptosis as described below.

In a separate experiment, B cells were magnetic bead-purified from DR4 Tg mice that had been immunized 21 days prior with chicken cII in incomplete Freund's adjuvant (Chondrex). Purified DR4^+ ^B cells were then cultured with an equal number of human cartilage glycoprotein-39 (HCgp39)-specific T hybridoma cells that are HLA-DR4-restricted [27,28]. The peptide, cII_259-273 _(irrelevant peptide for this hybridoma), was added to some cocultures as a control, whereas other cocultures contained the cognate HCgp39_263-275 _peptide (10 μg/mL). Neutralizing antibodies to human MHC class II (clone TDR31.1; Ancell Corporation, Bayport, MN, USA), mouse IL-10 (clone JES052A5; R&D Systems, Inc., Minneapolis, MN, USA), mouse FasL (clone 101626; R&D Systems, Inc.), and a rat IgG_1 _antibody (isotype control for anti-IL-10 and anti-FasL) were added at 10 μg/mL at the beginning of the culture. An agonist antibody recognizing mouse CD4 (clone GK1.5; eBioscience, Inc., San Diego, CA, USA) was used as a positive control for T-cell apoptosis. T hybridoma cells were collected at 48 hours of coculture and analyzed for T-cell apoptosis by three-color flow cytometry [29]. Cells were stained with anti-mouse CD4-PE, washed, and labeled with Annexin V-FITC and PI and then analyzed immediately on a Cytomics FC 500 flow cytometer. Gating was performed on CD4^+ ^and PI^- ^cells to exclude non-Th cells and cells that were already dead, and then labeling of Annexin-V-FITC was analyzed using FlowJo software.

### Cell cultures for analysis of cytokine production

Cell-free supernatants were collected on days 2 and 5 from cultures containing splenocytes (2 × 10^6 ^cells/mL) from CFA/cII immunized TCR Tg mice mixed with 10 μg/mL cII_259-273 _peptide. In a separate experiment, B cells were magnetic bead-purified from unimmunized DBA/1 mice and cultured in wells that had been precoated with an agonistic anti-mouse CD40 antibody or wells that were uncoated. After 24 hours of stimulation, cII_259-273 _peptide (10 μg/mL) was added to some wells, followed by the addition of neutralizing antibodies against mouse FasL or IL-10 (10 μg/mL). Splenocytes from unimmunized cII TCR Tg mice were depleted of CD19^+ ^B cells by magnetic bead separation and then added to control wells or wells containing B cells ± antigen. Cell-free culture supernatants were collected on days 2 and 5 and analyzed by sandwich enzyme-linked immunosorbent assay for the presence of IFNγ (BD Biosciences) and IL-17 (eBioscience, Inc.), respectively, using manufacturer protocols.

### Statistical analysis

Linear regression comparisons were performed using GraphPad Prism software (version 3; GraphPad Software, Inc., San Diego, CA, USA). Student *t *tests were performed on data sets in bar graphs and are reported as follows: one symbol indicates *P *< 0.05; two symbols, *P *< 0.02; and three symbols, *P *< 0.01. Designations for the symbols used for error comparisons in individual graphs are listed in the figure legends.

## Results

The initial aim of this study was to establish a potent arthritis model using a TCR Tg strain of mice specific for cII in the context of MHC class II I-A^q ^(DBA/1 background) [26]. An established immunization protocol for collagen-induced arthritis involving a single injection at the base of the tail of cII in CFA was used, and joint inflammation was noted for some mice beginning at day 16 after immunization and showed rapid progression to severe arthritis for some of the early responders (Table 1). However, a significant portion of the mice had no visible inflammation or had only mild arthritis by day 27 after immunization. Disease scores on day 27 ranged from 0 (nonarthritic) to 12 (highest possible), with good representation of all scores in between. Disease activity was independent of which cage the mice were housed in, and splenocytes from all mice responded to recall challenge with cII peptide *in vitro *by producing IL-17 or IFNγ or both, indicating that all had been successfully immunized. The broad range of disease activity suggested that there were individual differences in the immune responses to immunization that could not be explained solely by genetic or environmental factors.

**Table 1:** Arthritis scores of type II collagen/complete Freund's adjuvant-immunized T-cell receptor transgenic mice and total splenocyte counts at day 28

**Mouse^a^**	**Cage**	**Day 16^b^**	**Day 20**	**Day 23**	**Day 27**	**Final score**	**Category**	**Total number of splenocytes**
1	1	0,0,0,0	0,0,1,2	3,0,3,3	3,0,3,3	9	Severe	8.00 × 10^7^
3	1	0,0,0,0	0,0,0,1	0,1,0,0	3,3,0,0	6	Moderate	1.66 × 10^8^
4	1	3,0,1,0	3,3,1,1	3,3,3,3	3,3,3,3	12	Severe	8.72 × 10^7^
5	2	0,0,0,0	0,0,0,0	0,0,0,0	0,0,0,0	0	No arthritis	1.47 × 10^8^
6	2	0,0,0,0	0,0,0,0	0,0,0,0	0,0,0,0	0	No arthritis	1.46 × 10^8^
7	3	0,0,0,0	0,3,1,1	0,3,3,3	0,3,3,3	9	Severe	1.05 × 10^8^
8	3	0,2,0,0	1,3,0,0	1,3,0,0	1,3,0,1	5	Moderate	1.66 × 10^8^
9	3	0,0,0,0	0,0,0,0	0,0,0,0	0,0,1,1	2	Mild	1.22 × 10^8^
10	4	0,0,0,0	0,0,0,0	1,2,0,0	0,2,1,2	5	Moderate	1.56 × 10^8^
11	4	0,0,0,0	0,0,0,0	3,0,1,2	3,0,1,3	7	Severe	5.52 × 10^7^
12	5	0,0,0,0	0,0,1,0	0,0,1,0	0,0,1,0	1	Mild	1.25 × 10^8^
13	5	0,0,0,0	0,0,0,0	1,1,0,1	2,1,0,2	5	Moderate	1.18 × 10^8^
14	5	0,0,0,0	0,0,0,0	0,0,0,0	0,0,0,0	0	No arthritis	7.12 × 10^7^
15	6	0,0,0,0	0,0,0,0	0,0,0,1	0,3,1,3	7	Severe	6.72 × 10^7^
16	6	0,0,0,0	0,0,0,0	0,0,0,0	0,0,0,0	0	No arthritis	8.32 × 10^7^
17	6	0,0,0,0	0,0,0,0	0,0,1,0	0,0,3,1	4	Moderate	8.00 × 10^7^
18	7	0,0,0,0	0,0,0,0	1,2,0,1	1,3,1,3	8	Severe	9.68 × 10^7^
19	7	0,0,0,0	0,0,0,0	0,3,0,0	0,3,3,3	9	Severe	6.40 × 10^7^
20	7	0,0,0,0	0,0,1,1	0,0,2,3	0,0,2,3	5	Moderate	8.72 × 10^7^

^a^Mouse #2 was sacrificed at day 20 due to malnourishment, which was believed to be related to a dental abnormality. This mouse had no signs of arthritis on day 20. ^b^Arthritis disease scoring is based on a 3-point scale for each paw: 0 = no inflammation, 1 = inflammation confined to digits, 2 = mild swelling of paw, and 3 = severe swelling encompassing paw, ankle, wrist, and/or knee joints. Scores shown for individual dates are in the following order: right fore paw, left fore paw, right hind paw, and left hind paw.

**Figure 1:**
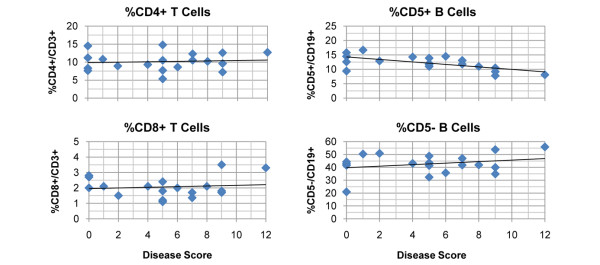
Reduced percentages of CD5^+ ^B lymphocytes in severe arthritis. Type II collagen/complete Freund's adjuvant (cII/CFA)-immunized, cII T-cell receptor transgenic mice (n = 19) were scored for arthritis severity on a 3-point scale for each paw and then euthanized on day 28 after immunization. Single-cell suspensions of splenic leukocytes were stained with antibodies as described in Materials and methods and analyzed by four-color flow cytometry. Percentages of CD4^+ ^and CD8^+ ^T cells (among CD3^+^/B220^- ^lymphocytes) and CD5^+ ^and CD5^- ^B cells (among CD19^+ ^lymphocytes) within the small nongranular lymphocyte gate were plotted for individual mice versus the final disease score. Only the percentage of CD5^+ ^B cells showed a significant decline as disease severity increased (R-squared = 0.4222, F = 11.69, *P *= 0.0035). Data are from a representative experiment of two experiments (n = 37 mice total).

Analysis of splenic lymphocyte populations from individual mice showed that the total number of white cells ranged from 5.5 × 10^7 ^to 1.7 × 10^8 ^cells per mouse, with a trend (that did not reach statistical significance) toward fewer total lymphocytes in the more severely arthritic mice (Table 1). Lymphocyte subset analysis revealed that the relative number of CD4^+^/CD3^+ ^T cells, CD8^+^/CD3^+ ^T cells, and CD5^-^/CD19^+ ^B cells was unchanged regardless of disease severity (Figure 1). However, a statistically significant reduction in CD5^+^/CD19^+ ^B cells was noted in the spleens correlating to increasing severity of inflammation (Figure 1).

**Figure 2:**
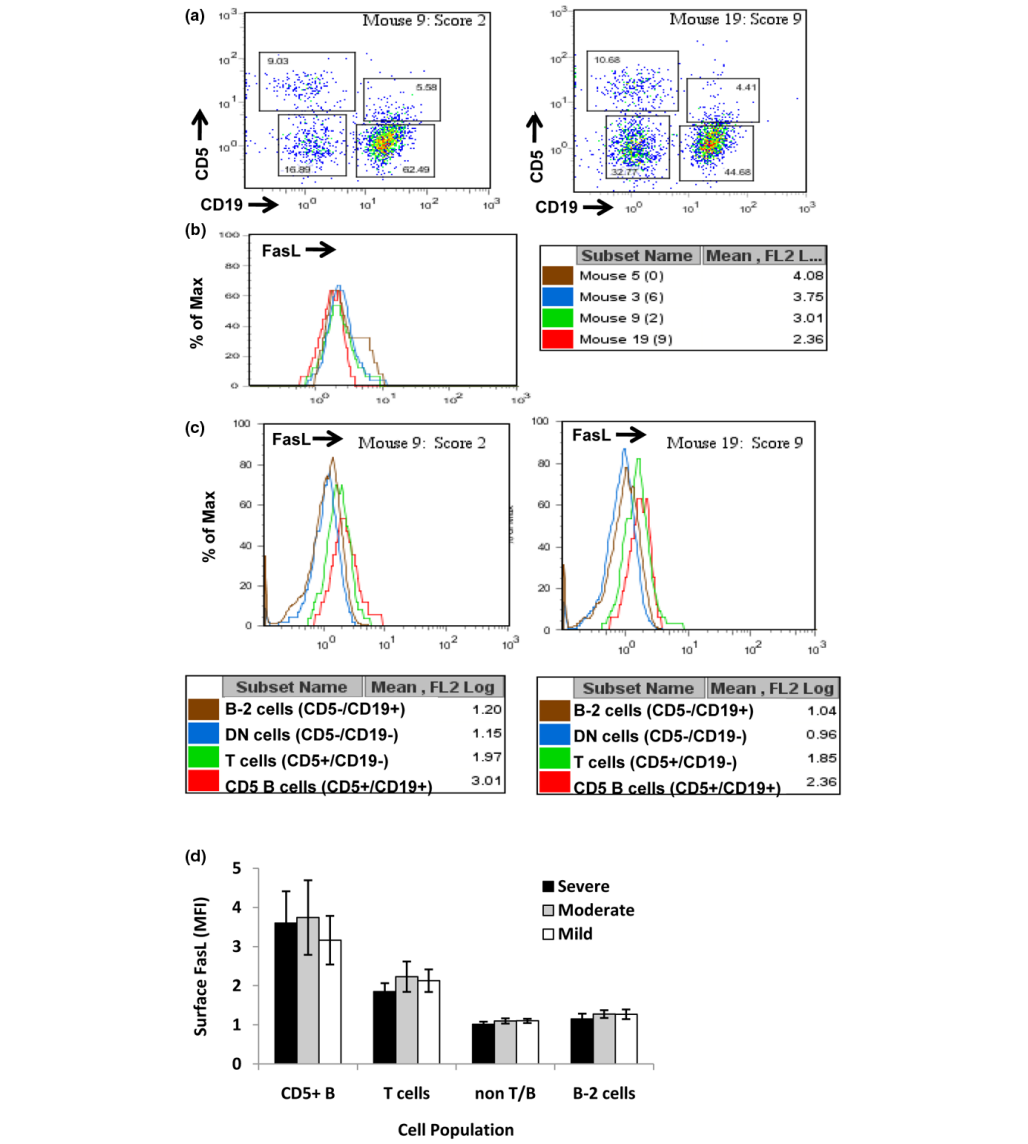
CD5^+ ^B cells express higher levels of Fas ligand (FasL) than other splenic populations. Freshly isolated splenocytes from type II collagen/complete Freund's adjuvant (cII/CFA)-immunized, cII T-cell receptor transgenic mice were stained with anti-CD19, anti-CD5, and anti-FasL antibodies as described in Materials and methods. **(a) **Cells were initially gated as small nongranular lymphocytes by forward scatter and side scatter criteria (not shown) and then categorized by staining with anti-CD19 and anti-CD5 as T cells (CD5^hi^/CD19^-^), CD5^+ ^B cells (CD5^+^/CD19^+^), B-2 cells (CD5^-^/CD19^+^), or double-negatives. **(b) **Comparison of FasL expression on CD5^+ ^B cells from four mice, with representative disease severity scores shown in parentheses. Numbers in the box are mean fluorescence intensity (MFI) values of FasL staining on CD5^+ ^B cells from the four mice. **(c) **Histograms and MFI values compared for lymphocyte subsets of two representative mice. **(d) **Average MFI values of FasL expression on gated splenic lymphocytes from mice with severe (n = 6), moderate (n = 6), and mild or no arthritis (n = 6).

Mouse CD5^+^/CD19^+ ^splenocytes were previously shown to be constitutively positive for expression of FasL and could be induced to express higher levels of FasL by exposure to antigens and the cytokines IL-4 and IL-10 [23]. Therefore, FasL expression was analyzed on splenic populations of the individual mice and compared with disease activity. Splenic lymphocytes were first gated on forward scatter and side scatter (not shown) and then subdivided on the basis of expression of CD5 and CD19 into four groups (Figure 2a). T cells (both CD4^+ ^and CD8^+^) are constitutively high expressers of CD5 and therefore comprise the CD5^+^/CD19^- ^fraction. CD5^+ ^B cells have lower expression of CD5 than T cells and were gated based on control samples that lacked T cells in order to exclude doublets of T and B cells from the analysis. FasL expression was highest on the CD5^+^/CD19^+ ^B-cell subset but was also detected at a lower intensity on CD5^+^/CD19^- ^T cells (Figure 2c, d). FasL expression varied slightly on CD5^+ ^B cells from individual mice (Figure 2b), but there were no significant differences in mean fluorescence intensity on CD5^+ ^B cells between mice with severe, moderate, or mild arthritis (Figure 2d and Table 1). Thus, differences were noted in the total number and percentage of FasL^+ ^CD5^+ ^'killer' B lymphocytes in severely arthritic mice but not in the intensity of FasL expression.

**Figure 3:**
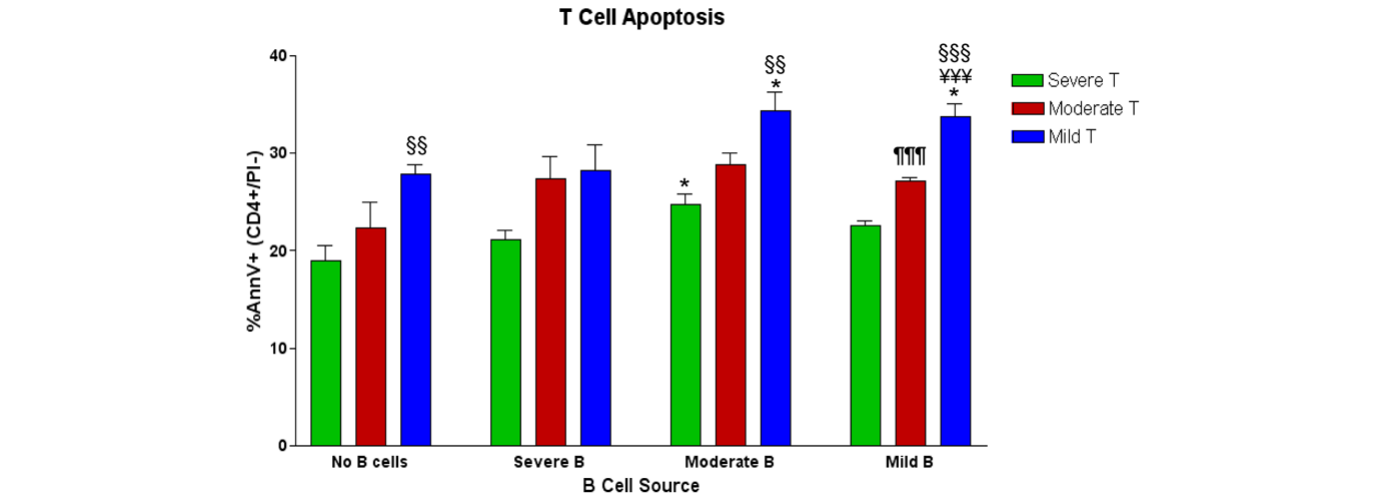
Antigen-stimulated T-cell death is dependent on disease severity and is mediated by B cells. CD19^+ ^B cells were depleted from pooled splenic cells of severely, moderately, and mildly arthritic mice (see Table 1 for groupings), leaving enriched populations of T cells. B-depleted cells from each group were cultured for 7 days with cII_257-273 _peptide and cocultured with purified B cells (1:1 ratio) from each group. Apoptosis was measured by Annexin V-based three-color flow cytometry as described in Materials and methods. Control T-cell apoptosis in parallel cultures without antigen ranged from 5.3% to 22.5%, followed a similar pattern between T-cell groups as shown above, was not stimulated by B cells, and was significantly lower than antigen-stimulated apoptosis for every combination. Data shown are mean ± standard error for triplicate samples. Statistical comparisons by Student *t *tests within groups are marked as ^§^mild T versus severe T, ^¥^mild T versus moderate T, and ^¶^moderate T versus severe T. Comparisons of B cell-mediated death are made to the corresponding culture without B cells and are designated with an asterisk. One symbol = *P *< 0.05, two symbols = *P *< 0.02, three symbols = *P *< 0.01. AnnV, Annexin V; PI, propidium iodide.

To determine the relative importance of killer B cells to T-cell apoptosis, splenocytes from six mice in each severity group were pooled, and then CD19^+ ^and CD19^- ^cell populations were isolated by magnetic bead separation. Cells were then recombined at a 1:1 ratio in the presence or absence of cognate antigen for 7 days and analyzed for T-cell apoptosis (Figure 3). Notably, T cells from severely arthritic mice had lower baseline apoptosis than those from mildly arthritic mice and were more resistant to the presence of B cells. Despite the already high baseline T-cell apoptosis observed in the B cell-depleted culture from mildly arthritic mice, the addition of B cells from moderately and mildly arthritic mice, but not severely arthritic mice, induced a significant increase in T-cell apoptosis (Figure 3).

**Figure 4:**
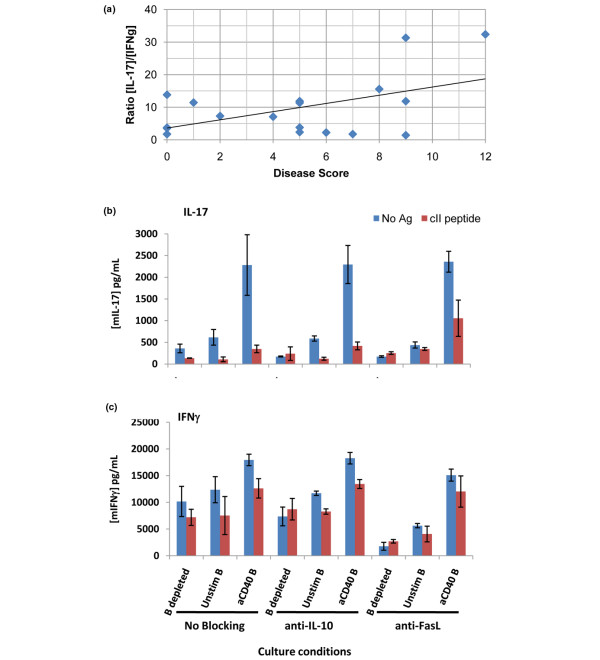
Interleukin-17/interferon-gamma (IL-17/IFNγ) ratio increases with disease severity and dependence on Fas ligand. **(a) **Single-cell suspensions of splenocytes from individual mice were cultured in the presence of 10 μg/mL of cII_259-273 _peptide. Culture supernatants were collected on days 2 and 5 and tested for IFNγ and IL-17, respectively, by enzyme-linked immunosorbent assay (ELISA) as described in Materials and methods. The ratio of antigen-stimulated IL-17 to IFNγ was determined for each mouse and plotted against the disease score for that mouse. Linear regression was performed using GraphPad Prism software and showed a significant correlation between a higher IL-17/IFNγ ratio as disease severity increased (R-squared = 0.2539, F = 5.444, *P *= 0.033). **(b-c) **Splenic CD19^+ ^B cells were magnetic bead-purified from naïve DBA/1 mice and cultured overnight in the presence or absence of plate-bound anti-mouse CD40 antibody. After 24 hours of culture, cII peptide was added to indicated samples, followed by the addition of splenocytes from naïve cII T-cell receptor transgenic mice that had been depleted of CD19^+ ^cells by magnetic bead separation. Supernatants were collected 5 days after the addition of B-depleted cells, and IL-17 (b) and IFNγ (c) levels were determined by ELISA. Data shown are mean ± standard deviation of triplicate samples. Ag, antigen; cII, type II collagen; FasL, Fas ligand.

Several lines of evidence have suggested that arthritis in mouse models is mediated through production of IL-17 by Th17 cells and is negatively regulated by the presence of IFNγ [6,8,9,30]. To determine whether the balance of these two cytokines might be shifted in individual mice, unseparated splenocytes from the immunized TCR Tg mice were cultured with antigen and tested for the release of cytokines into the culture supernatants. As noted above, splenocytes from all mice responded to antigen with production of IL-17 or IFNγ or both cytokines in these cultures, indicating successful immunization. However, comparison of the optimal production of IFNγ (day 2) and IL-17 (day 5) revealed a significant increase in the ratio of IL-17 to IFNγ in relation to increasing disease severity (Figure 4a).

Relatively little is known about the sensitivity of Th17 cells to apoptosis, particularly that mediated by killer B cells. As shown in Figures 4b and 4c, prestimulation of splenic B cells through CD40 led to a phenotype that was inductive of cytokines from naïve T cells of TCR Tg mice in the absence of cognate antigen but highly suppressive of IL-17 when antigen was present. This suggested that IL-17 production was tightly regulated by B cells through an antigen-dependent mechanism. Neutralization of FasL, but not IL-10, restored a significant proportion of the IL-17 production that was suppressed in the antigen-dependent coculture (Figure 4b). IFNγ production in the same cultures was less dependent on the blockade of FasL, although reduced IFNγ in the B cell-depleted culture in which anti-FasL antibody was present suggested that Fas/FasL interactions may actually stimulate IFNγ in some circumstances (Figure 4c).

**Figure 5:**
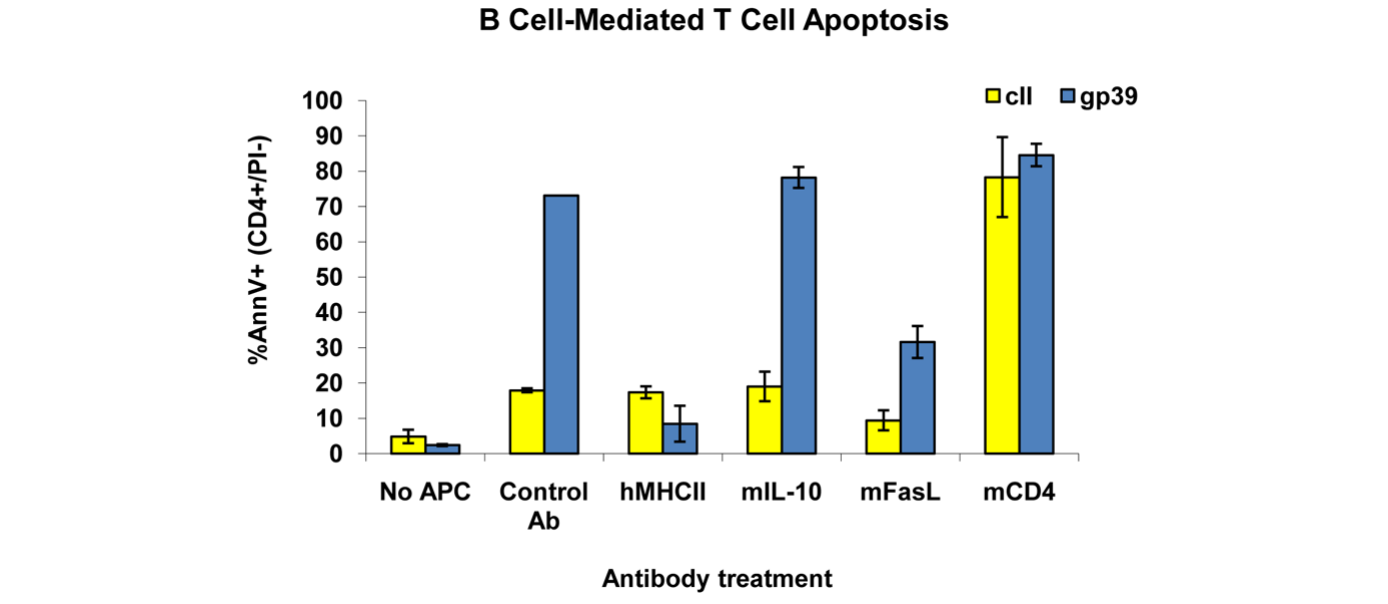
Peptide-specific and Fas ligand-dependent killing of T hybridoma cells by purified B cells. Splenic CD19^+ ^B cells were magnetic bead-purified from incomplete Freund's adjuvant/type II collagen (cII)-immunized human major histocompatibility complex (MHC) class II DR4 transgenic mice. Purified B cells (> 98% pure) were pulsed with peptides cII_259-273 _or HCgp39_263-275 _and then cultured with neutralizing antibodies against human MHC class II, mouse interleukin-10 (IL-10), or mouse Fas ligand. An isotype control antibody (rat IgG_1_) and an agonist anti-mouse CD4 antibody, used as a positive control for induction of T-cell apoptosis, were added to some wells as controls. One hour after the addition of antibodies, DR4-restricted, gp39-specific, T hybridoma cells were added at a 1:1 ratio to the original number of B cells. Cells were collected 24 hours later and analyzed for T-cell apoptosis by Annexin V-based, three-color flow cytometry. Data shown are mean ± standard deviation of triplicate samples. Ab, antibody; AnnV, Annexin V; APC, antigen-presenting cell; DR4, human class II major histocompatibility complex DRB1*0401; PI, propidium iodide.

To further demonstrate the ability of purified B cells to participate in antigen-specific T-cell death, coculture experiments were performed using a T-cell hybridoma cell line that recognizes the arthritis-associated peptide from HCgp39, presented by human HLA-DR4 [27,28]. As shown in Figure 5, T hybridoma cells by themselves do not undergo apoptosis in the presence of HCgp39 peptide. However, when purified splenic B cells from immunized DR4 Tg mice were added to the culture, antigen-dependent T-cell apoptosis occurred (control antibody condition). Neutralizing antibodies to MHC class II or FasL, but not anti-IL-10 or control antibodies, significantly decreased T-cell apoptosis, indicating that the B cells killed T cells through an antigen-specific and FasL-dependent mechanism.

## Discussion

Elimination of antigen-specific T cells is a possible approach to treatment of arthritis and other autoimmune diseases. Previous studies have shown that FasL^+ ^'killer' B cells occur naturally in mice and humans and can regulate the immune response [22]. In the present study, Tg mice carrying a TCR transgene specific for a cII peptide varied widely in the severity of joint inflammation following immunization with cII. This variability suggested that analysis of individual mice could lead to clues about the mechanisms controlling arthritis severity.

Various trivial explanations of this variability were excluded. Immunization of all 19 mice shown in Table 1 was performed on the same day by the same person with one antigen preparation. All mice showed evidence of successful immunization. A subsequent experiment with 18 additional mice yielded similar results (data not shown). Differences in disease severity were detected between mice in the same cage and from the same litter, suggesting that age, genetics, and environmental factors were not the major determinants of disease severity.

Differences in arthritis severity correlated with a reduction in the percentage of CD5^+^/CD19^+ ^splenic B cells but not CD5^-^/CD19^+ ^B cells or T cells. The trend toward fewer total lymphocytes in the spleens of severely arthritic mice indicates that the absolute number of CD5^+ ^B cells was also reduced with increasing arthritis severity. This result supports the hypothesis that CD5^+ ^B cells can regulate the severity of T cell-driven arthritis and prompted an analysis of the mechanisms by which this might occur.

Previous studies have shown that splenic CD5^+ ^B cells expressed FasL and could induce CD4^+ ^T-cell death. In the present study, CD5^+^/CD19^+ ^B cells were indeed the highest expressers of FasL among splenic lymphocytes in all mice. The mean fluorescence intensity of FasL staining was not significantly different on CD5^+ ^B cells from severely arthritic mice compared with moderately, mildly, or nonarthritic mice. This result contrasts with previous studies in a highly polarized Th2 system, in which higher expression of FasL on CD5^+ ^B cells was a mechanism for decreased inflammation [23]. Thus, the present data suggest that an insufficient number of killer B cells, rather than a decrease in FasL expression or functional killer activity by CD5^+ ^B cells, could be responsible for reduced immune regulation in severely arthritic mice.

To determine whether arthritis severity could be directly linked to killer function of B cells, coculture experiments were performed in which B cells were first removed from whole splenocyte preparations and then added back with or without antigen. T cells from severely arthritic mice were resistant to apoptosis compared with T cells from mice with less severe arthritis. B cells were capable of inducing T-cell apoptosis in an antigen-dependent and disease severity-dependent manner. These data suggested that the relatively small differences in the number of FasL^+ ^B cells in the cocultures played a significant role in directing T-cell death. An implication of these findings is that interventions that would reduce resistance of T cells to apoptosis and/or increase the number of FasL^+ ^CD5^+ ^B cells could tip the balance toward regaining immune tolerance.

The role of CD5^+ ^B cells in RA has been debated for many years. CD5^+ ^B cells are capable of producing autoreactive antibodies, including rheumatoid factor (RF) [31-33]. However, the relative contributions of CD5^+ ^and CD5^- ^B cells to RA pathogenesis remain elusive [34]. Some of the initial investigations comparing CD5^+ ^B cells in the circulation of RA patients and their nonarthritic first-degree relatives did not show significant differences, whereas other studies found not only significantly elevated CD5^+ ^B-cell levels in the blood of arthritic individuals, but also a correlation between the higher number of CD5^+ ^B cells and higher titers of IgM RF [35-37]. In another study, the relative number of IgG RF^+ ^CD5^+ ^B cells was reduced in arthritis patients whereas the number of IgG RF^+ ^CD5^- ^B cells was elevated [38]. Yet more confusing are the findings that elevated CD5^+ ^B-cell levels in peripheral blood are associated with both earlier onset of and remission of RA [39,40]. Perhaps the present findings of FasL expression on CD5^+ ^B cells and the inverse correlation between total numbers of splenic CD5^+ ^B cells and arthritis severity in mice can shed new light on these apparent contradictions.

If a normal function of CD5^+ ^B cells is to express FasL and regulate immune reactions through induction of T-cell apoptosis, then the specificity of CD5^+ ^B cells for autoantigens could be a mechanism leading to peripheral tolerance of autoreactive T cells. Uptake of autoantigens by CD5^+ ^B cells and subsequent presentation to T cells by cells that coexpress FasL would lead to selective elimination of the autoreactive T cells and protection from autoimmunity. However, if an individual had a defect in the number of CD5^+ ^B cells, in the expression of FasL by CD5^+ ^B cells, or in the T-cell response to death signals, then this mechanism of peripheral tolerance could be ineffective, and interaction of CD5^+ ^B cells with T cells might lead to exacerbation of disease by increasing the T-cell response to autoantigens.

Cocultures of naïve T cells with activated B cells in the presence of antigen and neutralizing antibody to FasL showed that B cells could preferentially regulate the production of IL-17 versus IFNγ in a FasL-dependent manner. Surprisingly, significant production of IL-17 was elicited by coculture of naïve T cells with anti-CD40-activated purified B cells when antigen was not present. In contrast to its role in antigen-dependent interactions, FasL did not appear to be regulating the production of IL-17 in the absence of antigen. This suggests that there may be antigen-independent interactions occurring between T- and B-cell populations that drive Th17 cell activity, a finding that may have implications concerning the role of activated B cells interacting with 'bystander' T cells.

Interestingly, blockade of IL-10 in these cocultures had no effect on the production of IL-17 or IFNγ. Cytokine production by B cells has received increasing attention over the past several years [41-44]. Among the cytokines produced by B cells, IL-6 and IL-10 are of particular interest because of their reported production by CD5^+ ^B cells and their respective pro- and anti-inflammatory effects in arthritis models [45,46]. Intracellular cytokine staining of freshly isolated splenocytes from immunized cII TCR Tg mice demonstrated that IL-10^+ ^subsets were found within both CD4^+ ^cells and CD5^+ ^B cells (data not shown). We found a significant decrease in the percentage of IL-10^+ ^CD5^+ ^B cells corresponding to increasing disease severity, which was not evident in the IL-10^+ ^or FoxP3^+ ^Treg cell subset (data not shown).

The ability of B cells to regulate immune responses through expression of FasL and induction of cell death of T cells is a fairly new concept [22]. Although antigen-dependent T-cell death has been demonstrated using purified CD5^+ ^B cells in the murine model of *Schistosoma mansoni *infection [23], the present report is the first to show that T-cell death could be stimulated using a peptide derived from an arthritis-associated autoantigenic peptide. Death induction was highly dependent on B cells, specific antigen, and MHC and was at least partially dependent on FasL. This suggests that killer B cells may be an effective mechanism to target antigen-specific T cells to undergo apoptosis.

At present, it is unclear whether CD5^+ ^B cells play a similar role in immune regulation in human patients. FasL expression has been detected on human B cells in CD5^+ ^B-CLL lines [47] but has never been directly studied on nonmalignant CD5^+ ^B cells in humans. Recent studies of B-cell depletion in RA patients have shown that there are clear therapeutic benefits to eliminating B cells, presumably by eliminating pathogenic B cells that may contribute to inflammation through antigen presentation to autoreactive T cells, proinflammatory cytokine production, and/or production of autoantibodies [48,49]. Although the present study might seem to contradict the benefit of B-cell depletion therapy, evidence in mouse models suggests that CD5^+ ^B cells may be protected from depletion mediated by anti-CD20 antibodies, and evidence from the anti-CD20 antibody trials in humans demonstrates that many of the earliest B cells that repopulate these patients express CD5 [50,51]. It will be interesting to determine whether the repopulating CD5^+ ^B cells also preferentially express FasL and to study immunoregulatory effects of these cells on human T lymphocytes.

## Conclusions

The present findings suggest a novel regulatory role for CD5^+ ^B cells on disease severity in a murine model of arthritis. The dependency of B cell-mediated killing on antigen specificity implies that FasL^+ ^B cells might be useful to eliminate arthritogenic T cells specifically, without harming other T cells that are necessary for defense against infections or neoplasm. If killer/regulatory B cells are shown to be important in controlling arthritic inflammation in humans, attractive new goals in the treatment of RA could include the enhancement of their killer activity or stimulation of their repopulation after B-cell depletion.

## Abbreviations

APC: antigen-presenting cell; CFA: complete Freund's adjuvant; cII: type II collagen; DR4: human class II major histocompatibility complex DRB1*0401; FasL: Fas ligand; FITC: fluorescein isothiocyanate; HCgp39: 39-kilodalton human cartilage glycoprotein; IFNγ: interferon-gamma; IL: interleukin; MHC: major histocompatibility complex; PBS: phosphate-buffered saline; PE: phycoerythrin; PECy7: phycoerythrin cychrome 7 conjugate; PI: propidium iodide; RA: rheumatoid arthritis; RF: rheumatoid factor; TCR: T-cell receptor; Tg: transgenic; Th: CD4^+ ^T helper; Treg: regulatory T.

## Competing interests

SKL declares that he has no competing interests relevant to this manuscript. DAF has received research and salary support from Genentech (South San Francisco, CA, USA). Genentech did not provide direct funding for this project or its publication.

## Authors' contributions

SKL was the main intellectual contributor to the design, execution, interpretation, and communication of this study. DAF provided substantive intellectual contributions related to study design and interpretation and was involved in the drafting of the manuscript. Both authors read and approved the final manuscript.

## Authors' information

SKL is a Research Assistant Professor in the Division of Rheumatology of the University of Michigan, Medical School, Department of Internal Medicine. He received his Ph.D. from Wayne State University in 2001, having studied T-cell apoptosis in the schistosome worm infection model. He has authored or co-authored 22 journal articles, including three invited reviews focused on Th17 cells and autoimmunity and a recently published review on killer B lymphocytes. DAF is Professor of Internal Medicine and Chief of the Division of Rheumatology at the University of Michigan Medical School. He received his M.D. from Harvard Medical School in 1978 and received clinical and research training at Brigham and Women's Hospital (Boston, MA, USA). He is author or co-author of over 100 peer-reviewed papers, six of them co-authored with SKL. He is a member of the American Society for Clinical Investigation and the Association of American Physicians and is a past president of the American College of Rheumatology.
